# Perceptions and knowledge of school management teams about non-communicable diseases and strategies to prevent them

**DOI:** 10.4102/hsag.v27i0.1781

**Published:** 2022-02-11

**Authors:** Sibusiso C. Nomatshila, Teke R. Apalata, Sikhumbuzo A. Mabunda

**Affiliations:** 1 Department of Public Health, Faculty of Health Sciences, Walter Sisulu University, Mthatha, South Africa; 2 Department of Laboratory Medicine, Faculty of Health Sciences, Walter Sisulu University, Mthatha, South Africa; 3 The George Institute for Global Health, University of New South Wales, Sydney, Australia

**Keywords:** management, non-communicable diseases, prevention, policy, schools

## Abstract

**Background:**

In 2016, non-communicable diseases (NCDs) were reported to be responsible for 41 million of the world’s 57 million deaths. These deaths were reported to be associated with modifiable lifestyle behaviours, such as tobacco smoking, poor physical activity and diets of poor nutritional value. There could be a knowledge gap on NCD risk factors amongst non-health professionals. Knowledge of NCDs is, therefore, important for the implementation of preventive measures to onset of NCDs.

**Aim:**

This study aimed at describing perceptions and knowledge of school management teams about NCDs and strategies to prevent them.

**Setting:**

This study was conducted in Mt Frere, South Africa.

**Methods:**

This explorative qualitative study using a phenomenological data collection approach was conducted amongst purposively selected school authorities in 2016–2017 to understand their perceptions and knowledge about NCDs and what can be performed to prevent them. Two focus group discussions (FGDs) were conducted using open-ended and unstructured questions guided by interview schedule. Tesch’s eight phases of thematic analysis approach was used to analyse narrative data resulting in two main themes and nine subthemes.

**Results:**

Two themes (understanding and prevention of NCDs, and control measures for NCDs) and nine sub-themes emerged from the data analysis. Inconsistent description of NCDs, its causes and controls were identified amongst school management teams in the FGD. Diet, poverty, societal factors, gaps between decision makers and communities, and poor policy implementation were identified by participants as major issues in the development of NCDs.

**Conclusion:**

There was no adequate knowledge on NCDs amongst the school management team participants. Improved visibility of health promotion personnel is needed to ensure community empowerment on NCDs prevention.

**Contribution:**

The findings in this study will help in closing the gaps in the implementation of preventive health services for NCDs within school health.

## Introduction

The prevalence of diseases associated with the heart, type 2 diabetes mellitus, some cancers and long-term infections of the respiratory system was reported as the primary cause of death globally in the past few decades (Habib & Saha 2010; Horton 2013; Nojilana et al. 2016, Solomons, Kruger & Puoane, 2017; Yuyun et al. 2020). In the developed countries, it is reported that non-communicable diseases (NCDs), especially cardiovascular diseases (CVDs), were responsible for high rates of morbidity and mortality, resulting in 90% of all deaths (Yuyun et al. 2020). According to the literature in 2010, NCDs were responsible for more than 63% of deaths globally (Alwan 2011; Terzic & Waldman 2011; Wagner & Brath, 2012). NCDs were responsible for 68% (38 million) of all global deaths registered in 2014 (World Health Organization [WHO] 2018a). In 2016 alone, NCDs were reported by the WHO as being responsible for 71% (41 million) of the world’s 57 million deaths (WHO 2018b). This clearly shows an increasing trajectory of 5% between 2010 and 2014, and an additional 3% between 2014 and 2016 of all deaths associated with NCDs (WHO 2014, 2018a, 2018b). Deaths attributed to NCDs are projected to increase to 75% of all deaths by 2030 (Wang & Wang 2020). Although for the past few decades, NCDs had been classified as diseases of the first world countries, these diseases had recently been showing an alarming rise in the low- and middle-income countries (Wagner & Brath, 2012). About 2.6 million (or 35%) of deaths reported in sub-Saharan Africa (SSA) are as a result of NCDs, and thus, NCDs are considered as the second most common cause of death in the region (Yuyun et al. 2020). According to the WHO, in South Africa, NCDs accounted for 51% (269 000) of all deaths (WHO 2018a). In recent decade, CVDs, one of NCDs and a disease of lifestyle, are reported to have accounted for 13% of all deaths in SSA (Yuyun et al. 2020).

The risk factors for cardiovascular disease include modifiable lifestyle behaviours, such as tobacco smoking, poor physical activity, diets of poor nutritional value, presence of hypertension, diabetes and obesity (Afshin et al. 2019; Price et al. 2018; Tibazarwa et al. 2009). In order for people to pursue healthy lifestyle changes, there is an important role played by perception and knowledge as health and healthy lifestyle rely on what is perceived as the most conventional way of life (Sridhar & Madhu 2002). Knowledge and perceptions could be better understood through the use of the health belief model (HBM), which indicates that one’s opinion of chances of getting NCDs would play an important role in determining their health behaviour (Glanz, Rimer & Viswanath 2008). Glanz et al. (2008) further stated that an individual’s opinion of the level of seriousness of NCDs and its consequences could guide their decision making towards healthy lifestyle.

Healthy lifestyles were described as generational health behaviours, which have led to the increase in NCDs, particularly during mid-to-late adulthood, as a consequence of modifiable risk factors that are generally adopted earlier in life (Peer et al. 2013, Spires et al. 2016).

Holdsworth et al. (2006) acknowledged contribution of diet in development of NCDs and recommended nutrition education, highlighting the benefits of using vegetables and fruits in the prevention of NCDs. The WHO asserts that in Africa, the rate of NCDs amongst the population can be attributed to poor knowledge and awareness of healthy lifestyle behaviours (WHO 2003). Bensley and Brookins-Fisher (2003) indicated that reduction of the impact of NCDs is based on a person’s capability to change his or her behaviour by having the knowledge and skills necessary to enact a desired behaviour.

In a study conducted by Holdsworth et al. (2006) amongst Senegalese women aged 20–50 years, it is transpired that majority of the studied population had awareness about effects of obesity on life expectancy. This study identified that knowledge of diabetes did not translate into understanding the consequences of its development as a NCD. However, age is an important factor in determining the level of knowledge about NCDs (Gamage & Jayawardana 2017; Kaba et al. 2017). It was revealed in a Bhutanese study that risky behaviour was strongly associated with inadequate health education programmes and expanding variance between knowledge, perceptions and health promotion practice on NCDs (Kohori-Segawa et al. 2020).

Effective health promotion behaviours have been reported to be disrupted by lack of knowledge and language proficiencies (Nagamatsu et al. 2020). The constructs of the HBM (perceived susceptibility, severity benefits and barriers, cues to action and self-efficacy) were reported to have influence on behaviours to prevent NCDs (Glanz et al. 2008; Nagamatsu et al. 2020). In 2014, a study was conducted in South Africa amongst young adults, and it was reported that middle-older adults perceived higher risk to NCDs compared with younger adults, which resulted in compromised preventative behaviours against NCDs assumed by young adult population (Kaba et al. 2017). Knowledge about NCDs would enable individuals to take better care for associated risk factors, thus changing the attitudes and perceptions towards NCDs. The purpose of this study was to describe perceptions and knowledge of school authorities about NCDs and strategies to prevent them. Even though we acknowledge that there are other lifestyle NCDs, including some cancers, the focus is specifically on CVD and diabetes mellitus as they are most commonly associated with diet and sedentary lifestyle.

## Methodology

### Study design

A phenomenological approach was used to collect data in this explorative, qualitative study, which was conducted to understand the perceptions and knowledge of school management teams (SMTs) about NCDs and what can be performed to prevent them.

### Setting

The study was conducted in Mt Frere (KwaBhaca) in the Eastern Cape province, South Africa. Mt Frere is a rural town geographically positioned in the Alfred Nzo district municipality, which is located in the north-eastern part of the Eastern Cape province. According to Stats (2011), Alfred Nzo district has a population of more than 5200 people. The community is categorised as having lack of resources and infrastructure. The area is predominantly occupied by the Black African population from different socio-economic backgrounds with substantial obesity burden (Micklesfield et al. 2013; Shisana et al. 2014).

### Population

Participants included SMT (principals, heads of academic departments, chairpersons of the School Governing Bodies [SGBs], teachers and other members of the SGBs). The chairperson of the SGB is a parent representative in the committee. These were all decision makers for schools and were considered essential participants for the two focus group discussions (FGDs) of 6–10 participants in each. Although these participants were not considered representative of the population of the decision makers at schools in the country, they represented school authorities in Mt Frere.

### Sampling procedure

This study assumed purposive sampling for FGDs. The FGDs with 6–10 participants aimed to determine knowledge and perception of NCDs risks factors. Inclusion criteria encapsulated all members of the SMT available on the day of FGDs. The SMT embraced a broader population of teachers and parents, who are the representatives of the community, which provided comprehensive and diversified knowledge and perceptions.

### Data collection

#### Focus group discussions

All participants were allowed to contribute to the FGDs that are conducted to explore the perceptions and knowledge of school authorities about NCDs and methods of preventing them. A total of two FGDs consisting of six participants in the first and 10 participants in the second focus group lasting for about 60 min each were conducted. The discussions were facilitated by an expert in the field of public health by asking open-ended and unstructured questions based on interview guide. All FGDs were conducted in isiXhosa, which was considered a local language that was commonly used. Discussions were recorded using an audio recorder. This was followed by verbatim transcription carried out by an experienced and fluent isiXhosa transcriber for analysis.

### Data analyses

The narrative data from unstructured interviews guided with listed questions were analysed using Tech’s eight phases thematic analysis approach as cited by Micklesfield and colleagues (Creswell & Creswell 2017). Immediately after data collection, FGD data were analysed. It is recommended that analysis of FGDs should commence immediately after FGDs have ended (Nagle & Williams 2013). Verbatim transcription of field data from audio tapes was also carried out. To avoid inaccurate interpretation of essential information from the FGDs, Plummer-D’Amato (2008) recommended that transcriptions should be performed as was spoken. The transcriptions in this study were written out from local language (isiXhosa) into English text and back to isiXhosa to confirm if the meaning is consistent with the response from the participant. The audio tape was played intermittently in order to accurately capture the content in the audio tapes. Data comprehensiveness was ensured by typing the field notes and integrating into the main transcripts all the topic-based content. The meaning and sense of the information from the transcripts were obtained through repetitive reading of the transcripts by the researcher. Categories with similar meanings were grouped together, and themes were then described to represent the clustered categories. Themes and sub-themes identified using this approach formed the evidence base from which this research drew its conclusions. Data were transcribed, coded and clustered; themes were developed and an independent coder was used to confirm the themes.

### Ethical considerations

The study was presented to the Department of Public Health in the Faculty of Health Sciences. The Human Research Ethics and Biosafety Committee of Walter Sisulu University was approached for ethical clearance (reference number: 070/15) and the Eastern Cape Departments of Health and Education were approached for permission.

Informed consent with full details of the study and reporting channels was obtained from the participants, which allowed the researcher to tape-record the voice of FGDs. Participants’ names were not recorded to maintain confidentiality. Research records were kept in a locked filing system and all electronic information was coded and secured using a password protected file. The identity of participants was not disclosed in any published material.

The researcher ensured that no harm was caused to the participants. Those who refused to consent were not prejudiced by their choice. Information shared by participants was kept confidential. Respect for the dignity, safety and well-being of participants was given a priority with full recognition of culture, language, beliefs, perceptions, educational level and customs of the participants.

The respondents had the right to participate or not to participate in the study, and none of them were coerced to participate in the study. Participants had the right to withdraw at any stage of the study and were not forced to answer all questions if they did not feel comfortable.

The study was conducted in a manner that had the best interest of the participants. Participants were treated with utmost respect. Where a gap in the interpretation and explanation of policies was identified, corrective explanations were later provided or referral was made.

The study was conducted outside normal academic periods for participants to enable educators to continue with their academic work without possibilities of disturbance. Participants were allowed to remain anonymous for recording and were not required to pay or be paid for participation in the study to ensure confidentiality and anonymity of the participants. This was performed with full understanding that their participation could help in the management of NCD risk factors in the general population without inflicting any harm to self and others.

### Measures to ensure trustworthiness

According to Merriam (1998), the qualitative investigator’s equivalent concept, that is, credibility, deals with the question, ‘how congruent are the findings with reality?’ Credibility is described by Korstjens and Moser (2017) as the confidence that can be afforded in the truthfulness of the research outcomes in determining the plausibility of such outcomes as obtained directly from the study population. This was carried out by extending the time spent with participants in ensuring consistency and better understanding of their responses on NCD risk factors. This encouraged open relations between researcher and respondents. Respondents were encouraged to own the research as a way of improving their skills and abilities in preventing NCDs risk factors. Use of tape recording, observations and note taking ensured that a variety of information was collected and aligned together to ensure accuracy in the recording of such information or responses. In qualitative studies, scope is obtained through prolonged engagement, whilst the depth of a discussion is obtained through persistent observation (Lincoln & Guba 1985).

Korstjens and Moser (2017) and Tracy (2010) explained transferability as not just conduct and experiences but also the context in which these behaviours and experiences can make sense to any ordinary person. Streubert (2007) suggested that transferability was understood to demonstrate the probability that the findings of the study gave meaning to others in a similar situation. Different categories for participants according to their positions and responsibilities have been reported to ensure transferability in the study, whilst their verbatim quotes are provided in data analysis. In this study, there was regular contact with the participants to obtain views, behaviours and experiences on transcripts.

Data saturation was achieved at the point when no new concepts were raised and there was repeated and consistent findings gathered from the FGDs. This is in line with the description of dependability (Lincoln, Lynham & Guba, 2011). Documents such as field notes taken during data collection, constructed ideas emanating from analysis processes and categorisation of data and recordings were kept by the researchers. Test-and-retest was used to confirm the correctness of the transcribed data.

## Results

A total of 16 SMT members from two schools in Mt Frere formed part of the FGDs. The first FGD had 10 participants, whilst the second had 6. The first FGD took 60 min and the second 70 min.

The results of this study guided by two themes and nine sub-themes are described in Table 1.

**TABLE 1:** Themes of the knowledge and perceptions on non-communicable diseases and preventive measures.

Theme	Subthemes
Understanding and prevention of NCDs	Death
	Diet
	Treatment
NCD control measures	Government
	Awareness
	Diet control
	Gardening
	Parental care
	School menu

NCDs, non-communicable diseases.

## Knowledge and perception of participants about non-communicable diseases

### Theme 1: Understanding and prevention of non-communicable diseases

#### Subtheme 1.1: Death

When participants in the FGD were asked what they understood about NCDs, one participant said:

‘We know them that they kill a person and also kill the communities.’ (Participant 1, FGD 2, parent)

The response that NCDs were the cause of death was common amongst the FGDs and understanding of how they could be prevented or how would the situation be turned around was reported to be necessary. The groups were asked if they thought NCDs were an important health issue that should be taken care of. The common responses amongst the participants were that:

‘They are important and they require attention.’ (Participant 1, FGD 1, parent)

#### Subtheme 1.2: Diet

The SMTs recognised that NCDs were important topic that required attention. This was based on the notion that participants understood key risk factors to the development of the NCDs with its primary prevention and said:

‘If you do not eat appropriately, you end up having them. As such, you need to pay particular attention to food you eat so that we prevent them.’ (Participant 2, FGD 1, educator)

One of the participants felt that poverty and food security could be blamed for poor adherence to treatment, whilst other participants felt that the impasse could be blamed for the escalation in the prevalence of NCDs in their communities and said:

‘The other person would have the treatment but is unable to take it because they do not have money to buy food she is supposed to eat. Maybe the person is a granny staying with grandchildren and has to buy food to accommodate everyone thus unable to have specific diet for herself.’ (Participant 2, FGD 2, parent)

#### Subtheme 1.3: Treatment

The role played by diet in the development of NCDs was supported by other participants who recognised and felt control of diet was essential. The participants further felt that adherence to treatment was another major challenge when they responded and said:

‘Some patients whilst having been told that they had those diseases, do not take their medicines, whilst some collected the medicine that will help them, put it there in the house and never use it.’ (Participant 3, FGD 2, parent)

## Strategies to prevent non-communicable diseases

### Theme 2: Non-communicable disease control measures

#### Subtheme 2.1: Government

Most of the population served by the schools were reported to be reliant on government social support grants including child support grants, old age grants and disability grants in that order. However, this source of income was thought of as being inadequate for maintenance of the household dietary needs and other amenities. This was therefore thought to be responsible for worsening malnutrition and NCDs. Comparisons of the government social support grants were even made with those of other countries:

‘If you could go to Brazil and compare their social assistance programme, you could find that there is a huge difference compared with South Africa. I can see that the grant does not help much because it is small and only increases by only few Rands per year.’ (Participant 3, FGD 2, parent)

In the process of identifying how communities would control the development and manage NCDs, participants were asked about what they thought could be done in preventing the NCDs in the communities and one participant felt government still had a major responsibility:

‘If government could help us. It is just that they sit in parliament and take decisions to be implemented but do not do follow-up on how awareness is conducted and explain to people about how that should be done.’ (Participant 5, FGD 2, educator)

With the effort of establishing the reasons behind poor visibility of health professionals responsible for cascading information and social transformation, one of the participants blamed the compensation of these employees and said:

‘Government needs to increase wages to those responsible.’ (Participant 3, FGD 1, parent)

#### Subtheme 2.2: Awareness

A participant from the FGD highlighted the importance of awareness for NCDs amongst parents and said:

‘Awareness is what is needed because if our communities were aware, even we as teachers as enlightened people would be aware before we went to children in classes. We would create awareness in our own communities and probably could have influence to counter the development of these diseases.’ (Participant 4, FGD 1, educator)

This was echoed by another participant who further explained the importance of their awareness about the disease risk factors and said:

‘If we are aware of dangers of fat cakes and others, even our children could be aware. We need to make communities aware so that we do away with things like these.’ (Participant 2, FGD 2, parent)

One of the SGB members supported the need to supervise what was fed to their children at homes and at all other environments including what they themselves were feeding their children during breakfast:

‘… although there is no alternative, with awareness and support, communities can be aware that soft porridge is not an ideal meal.’ (Participant 6, FGD 1, parent)

The challenge expressed by other FGD was that the problem was with children who seem not to prefer fruit as part of their daily diet. As such, so much food gets wasted within the school nutrition programme wherein children threw the fruit away after only one bite on it. One of the SMT members who participated in the FGD expressed difficulties in ways of dealing with NCDs risk factors such as diet and said:

‘There is difficulty everywhere. Although you educate a learner that fruit is good than chips but when you have given them a Rand, they know that they will be able to buy two packets of chips than an apple.’ (Participant 5, FGD 1, educator)

#### Subtheme 2.3: Diet control

The societal factors facilitated the continuance of the undesirable behaviour by the communities whilst they could not avoid it. Similarly, to this notion, one of the participants expressed the effects of the societal factors towards their health and said:

‘… I know that if I use salty food, that could kill me, but I don’t have a choice as my money or budget is too little for me to buy healthy foods.’ (Participant 1, FGD 2, parent)

‘… these children do not like to eat an apple. You find it thrown away around the school.’ (Participant 2, FGD 2, educator)

#### Subtheme 2.4: Gardening

The presence of gardens in schools was an initiative by participants. However, they felt that SMTs should not have been the people to facilitate or run them, but a special person be employed for them:

‘… there was a need for school garden, which was a very good initiative but its implementation was very poor given that there was no dedicated person responsible for it as such it added burden on teachers.’ (Participant 9, FGD 2, educator)

The effects and impact of the school gardens were expressed as phenomenal but needed to be refined. One of the participants also expressed the same sentiments and further supported the need and importance of both community and school gardens and said:

‘Schools and communities must have gardens and we must have a specific person responsible for that at school.’ (Participant 10, FGD 2, educator)

#### Subtheme 2.5: Parental care

Other participant supported the establishment of school and community gardens in support of children’s nutritional needs and expressed her observations by saying:

‘… we have parents who do not care. They sleep and never care to see their children off to school.’ (Participant 4, FGD 1, parent)

#### Subtheme 2.6: School menu

Participants were concerned about the level of concentration for their children at school, blaming it to skipping of breakfast. One participant felt that something else could be done within the National School Nutrition Programme (NSNP) and suggested that:

‘… there must be something for the morning including food handlers for the morning to prepare breakfast as our schools have high numbers of children, and food handlers can’t manage to prepare all these.’ (Participant 1, FGD 2, parent)

Participants described their strategy of managing the skipping of breakfast by suggesting that:

‘A child needs to go for morning prayers and leave for class with half a cup of milk, a boiled egg and at least two slices of bread with either margarine or peanut butter.’ (Participant 4, FGD 2, parent)

## Discussion

This study was conducted to explore the perceptions and knowledge of school authorities about NCDs and what could be done to prevent them. The WHO describes NCDs as diseases that are not passed from person to person (WHO 2018b). The study identified inconsistent description of NCDs amongst members of the SMT. Members of the SMT described the NCDs as diseases that killed. Although this description is categorised as ‘perceived severity’ under the Bandura’s HBM, it suggests a narrow understanding of the NCDs as all what was known was that they lead to death. Limited knowledge about a disease result in poor preventative measures and poor management of the disease (Kohori-Segawa et al. 2020). Causes and controls for NCDs were identified to be minimally understood or known by the SMTs. Limited understanding has a potential to yield undesirable health outcomes if the key people to child development were unable to adequately describe the diseases. It could not be expected that children and adolescents could pursue preventive behaviours of the NCDs if less was known about risk factors by their parents and teachers. Knowledge about the diseases could increase efficacy of an individual towards prevention and management of the disease. Gaps in knowledge were reported to be reflecting on the amount of information received by an individual and subsequently affected the use of such information for disease prevention as there are no perceived threats (Aribike et al. 2019).

The HBM for behaviour change by Bandura suggests that for an individual to change the behaviour, he or she goes through various steps that include perceived susceptibility to experiencing the condition, understanding of the severity of the condition once acquired, one’s belief of achieving something when changing behaviour and so on. After understanding risk factors for NCDs, the participants in the group felt susceptible to acquiring the NCDs and further understood that these diseases could lead to death as a severity of the consequences.

Diet was identified as a major issue in the development of NCDs. It could be observed in line with the findings by Pienaar (2015) who identified an increase in risk factors (obesity) in quantile 2 and 3 schools in South Africa between 2010 and 2013. This increase could be associated with the strengthening of the school nutrition programme and, essentially, what children ate during the day at school. Proper diet plays a significant role in control of diseases, including NCDs. This requires adequate financial resources including proper social support system. Family income or social support plays an important role in food availability and quality of food important for better health outcomes (Marmot & Bell 2019). Participants in FGDs felt social security support received played an important role in ensuring that household had adequate food and were able to buy good quality foods for their families. By understanding the role of diet in the reduction of NCDs, participants further felt that the issue of diet was complicated but it was exacerbated by poor adherence to treatment when one had already been diagnosed. This made people more susceptible to the onset of the diseases. Spires et al. (2016) cited that there was an upward trend in overweight and obesity owing to unhealthy food choices, which are NCD risk factors.

Poor adherence to treatment was blamed by the participants on negligence resulting from not taking medicines after being diagnosed. This suggested a combination of coexisting problems in the society with the problem of pharmacovigilance and efficacy that remained the challenge in the fight against the spread of the NCDs. This was also contrary to the understanding of the severity of NCDs as described by Bandura in the HBM. However, community knowledge of the disease genesis seemed to have an effect in the development and control of the diseases. In such situations, the decisions made by individuals, information given by the health personnel and community-based advocacy were suggested as progressive activities towards change of behaviour (Chigbu et al. 2013).

It was established by the participants that poverty levels in the communities were also contributing to both the development and poor management of NCD risk factors. It is reported that individuals from poor financial backgrounds were deprived of preventive health services such as screening, early diagnosis of diseases, access to life-changing health promotion messages, and successful treatment and therapy (Juma et al. 2019). The elderly people received their old age grants so as to take care of themselves but the situations in different homes were unfavourable and they were the main source of income in their respective families. The primary purpose for the old age grant was thus defeated. This suggested that efforts on the management of diet could be prolonged if the current plight of socioeconomic status was not reversed. The socio-economic status of a person was reported to be associated with severe outcomes and progression levels of NCDs risk factors amongst the elderly population (Stringhini et al. 2018).

As a strategy to manage socio-economic divides, a social security programme exists in South Africa. Use of grants in purposes beyond purported functions suggests that people could not help in improving dietary intake and did not contribute meaningfully to the prevention and management of NCD risk factors. As such, they required support through other programmes. The quality and quantity of food were highly compromised with low socioeconomic statuses of the families as was also suggested by Bouis, Eozenou and Rahman (2011). This understanding suggested that the South African social security system was not in line with other international counterparts with almost the same characteristics as South Africa’s.

Considering the situation and the state of social security, families and communities still had a responsibility to take care of their own health. Health promotion is defined in the Ottawa Charter as ‘the process of enabling people to increase control over and to improve their health’ and, as such, there was common interest in ensuring that people were not only waiting to be serviced by government but how would they service themselves.

Participants felt that there were clear gaps between decision makers and communities and policy implementation. Strategies and decisions taken were poorly communicated by relevant stakeholders to the communities and as such could not develop desirable health outcomes. It was discovered that awareness was not created amongst people, which decreased their efficacy and power to control their health including NCDs risk factors. Visibility and efficiency of the healthcare professionals responsible for the dissemination of information including quality health education was brought into question by these participants. This was also recommended by Kolbe-Alexander and Lambert (2013) who indicated that NCDs and their associated risk factors could be reduced through strong health promotion programmes.

There was a belief that when wages were improved, there would be improved visibility regardless of the availability or adequacy of such personnel. The participant felt that the wages given to the health professionals responsible for creating awareness and community empowerment were not adequate and were not encouraging that the said people do their work in a diligent and satisfying manner. This is in support of the findings of a 2012 study conducted in Ghana by Bonenberger et al. (2014). Personnel availability and satisfaction with their job were considered problems in the creation of awareness. If the already fewer personnel were not compensated satisfactorily, community empowerment or knowledge development programs would be compromised.

The desire for awareness was clearly determined by the participants across the FGD, which indicated that the level of community awareness was low and strategies employed in social marketing seemed to be facing challenges. In order to promote healthy lifestyles amongst young generation, there is a need to broaden health education and promotion or awareness spectrum with regard to NCDs and associated risk factors (Shivalli et al. 2012). Teachers were a strategic partner in creating awareness as they were in contact with hundreds of children in a day who could, in turn, be in contact with scores of people outside school environment. This supported the value of an empowered educator who would empower many communities through empowering a child. When the communities had been made aware of the dangers of foods children bought and brought to schools, they could be able to control NCDs and consequently, parents could not be the very same people who sold such foods to children. These could be controlled not only on the school environment but also in the community. Thus, the nation could be saved. Li et al. (2014) suggested that there was a strong need for unhealthy food restrictions in schools for the control of the development of NCDs risk factors.

Furthermore, the food children eat before going to school was also investigated. Participants revealed that children predominantly had a breakfast of maize-based porridge, prepared using refined and sifted maize meal, which was considered to be of high glycaemic index with high calories. Knowledge of what formulated good breakfast for children and adolescents before they went for school was expressed as a challenge in Mt Frere, Eastern Cape. How children had been nurtured at home and how diets at different households were structured reflected in the children’s eating habits. There is a reported association between parenting practices and children’s behaviour (Gubbels et al. 2011). Participants felt that parents did not pay any particular attention to what happened at home before their children left for school in the mornings. This was also explained in the context of skipping breakfast and reliance on school nutrition programme by parents. Although some families did not actually have something to give to their children in the morning, participants expressed their discomfort with how parents prioritised their children’s eating patterns. This expression created an understanding that the importance of breakfast for children needed attention. Eating breakfast did not only play an important role in mental health of children but improved behavioural pattern compared with those who skipped breakfast (Ahadi et al. 2016; Johansen, Rasmussen & Madsen 2006; Lien 2007). Participants understood that children came from households with high poverty rate and some truthfully had nothing to take for breakfast. Restructuring and expansion of the programme was identified as a need which, when addressed, could not only address reduction of effects of NCDs risk factors but also contribute meaningfully to academic outcomes. O’Neil et al. suggested that healthy body weight and overall health were benefits of eating breakfast and proper dietary intake, which also resulted in reduced risk of NCDs (O’Neil et al. 2014).

O’Neil et al. (2014) further suggested that regular breakfast eating results in improved cognitive development, thus leading to better academic performance. The same sentiments were echoed by other participants who suggested a new strategy in the implementation of the NSNP and said this was associated with socioeconomic imbalances that exposed children and adolescents in Mt Frere to challenges posed by the coexistence of numerous risk factors and poverty. It was reflected in the FGD that even when the communities were aware of the dangers of having high calorie foods, there were no alternatives as the communities were living under the poverty line. Even if they were eager to change, financial situations could be the barrier. This is further supported by Bouis et al. (2011) when they suggested that family income resulted in reduced dietary quality. The HBM for individual behaviour change expresses that one of the factors determining behaviour change is barriers to the implementation of the change (Skinner, Tiro & Champion 2015). This expression by the HBM is reflected in the expression by participants in the study. When that was read together with the social-ecological model, which expresses that one’s behaviour is influenced by intrapersonal, interpersonal, community and societal factors, wherein in the societal factors encapsulate socioeconomic state. Therefore, one can deduce that socioeconomic imbalances were contributory factors to the increase of the risk factors of NCDs.

The effects of societal factors seemed to be a common factor amongst challenges expressed in the FGDs. However, building from good lessons learnt and identifying cues to action towards reversal of the impasse, members had to identify what they thought could be done in addressing this. It transpired that there was no alternative to better health than to choose healthy eating and ensuring that children ate those lead to better health outcomes. Salty and high glycaemic foods were regarded as harmful for children and adolescents. As such, new ideas were considered imperative. One SMT member felt that the policy shifts created mixed feelings in management of risk factors.

The health promoting school policy was implemented at schools as part of the policy shift and it involved the establishment of the school gardens. This was viewed as an important strategy not only to address issues of poverty but also to contribute towards healthy eating aimed at reducing effects of NCDs. This innovation further supported the implementation of the food-based dietary guidelines, which stated that one must eat plenty of vegetables and fruit every day (Vorster, Badham & Venter 2013). Fruits and vegetables were essential in the prevention of the onset of the NCDs as they played a meaningful role in the control and management of overweight or obesity (Kopf et al. 2018).

## Limitations

Regardless of all endeavours for delimitations, there were limitations in this study. Firstly, the study only focused on schools where children from the Prospective Urban Rural Epidemiology (PURE) families were enrolled. It did not cover the entire Mt Frere population. In terms of the Department of Education’s regulations, parents and guardians from these families were eligible of being elected to be part of any governing structures in these schools. Secondly, even though parents are represented in the SMT, there could be a selection bias in those parents who are elected to be in the SGB/SMT. We, however, wanted to get their perceptions as community members and governance members’ representation was not our objective. Thirdly, the population of interest was only in a few schools in Mt Frere town. Subsequently, this study cannot be considered representative of the entire Eastern Cape province. However, its findings provide an acceptable picture of how the school management understood and perceived NCDs.

## Conclusion

School management team members who participated in this study have a strong feeling on the need for disseminating information on NCDs in the area to ensure that the NCDs are halted. People need to be made aware of the development and progression of the disease to strengthen one’s survival strategy, including the structuring of diet and how one pursues his or her health. The severity of the outcomes because of poor preventive measures necessitates stringent attention. The perception on how government system/programme operates suggests that there is no clarity about how things should be implemented. As such, there is a need to improve policy development and coordination towards reduction of the effects of NCDs in communities. Visibility and efficiency of the healthcare professionals responsible for the dissemination of information, including quality health education, remain the area of concern and require strengthening.
